# Editorial: Women in head and neck cancer 2021

**DOI:** 10.3389/fonc.2022.1024210

**Published:** 2022-12-13

**Authors:** Panagiota Economopoulou, Ioannis Kotsantis, Amanda Psyrri

**Affiliations:** Section of Medical Oncology, Department of Internal Medicine, Faculty of Medicine, National and Kapodistrian University of Athens, Attikon University Hospital, Athens, Greece

**Keywords:** immunotherapy, head and neck cancer, HPV, salivary gland tumors, biomarkers

In the Research Topic that accompanies this editorial, seventeen articles were included that cover various topics in head and neck cancer, such as systemic therapy, immunotherapy, novel therapeutic targets, analysis of surgical techniques and their complications, radiomics, prognostic and predictive biomarkers. This special issue showcased important contribution from female colleagues in the head and neck oncology.

As demonstrated in the Keynote 048 phase III trial (1), Programmed Death 1 (PD1) checkpoint inhibitor pembrolizumab, either as monotherapy or in combination with chemotherapy, increased overall survival in recurrent metastatic (R/M) squamous cell carcinoma of the head and neck (HNSCC) for patients whose tumors exhibit high expression of PDligand-1 (PD-L1), as defined by a combined positive score (CPS) ≥ 1. On the contrary, for tumors with PD-L1 CPS<1, the EXTREME regimen was shown to be equivalent to chemo/immunotherapy combination (2). In the current Research Topic, Lubbers et al. presented real-world data from a retrospective analysis that included 124 patients with R/M HNSCC before the approval of immunotherapy as first line therapy. Further supporting the results of the EXTREME trial (3), it was shown that the EXTREME regimen was superior to all other chemotherapy regimens in the 1^st^ line setting. Thus, in patients with PDL1 CPS score <1 or who have major contraindications for immunotherapy administration, EXTREME remains the standard of care. Nevertheless, there is an unmet need for more chemotherapy options for patients with severe comorbidities who are particularly frail and unfit for EXTREME. In this context, Carinato et al. presented retrospective data from 60 patients who were treated with weekly carboplatin, paclitaxel and cetuximab and found comparable survival outcomes with the standard EXTREME regimen.

Despite a remarkable progress in the treatment of head and neck tumors, a minority of patients derive actual benefit from approved therapies, and the only clinically relevant predictive biomarker is PD-L1 CPS score. Current studies focus on the development of clinical and molecular predictive or prognostic biomarkers that could be associated with clinical aggressiveness, response to therapy or clinical outcomes. Arribas et al. showed that low skeletal muscle index (SMI) at baseline, that was calculated *via* SliceOmatic software using a CT scan with the 3^rd^ lumbar vertebra as a reference point, might have a negative impact on survival in patients receiving immune checkpoint inhibitors. On the other hand, Giunco et al. evaluated surgical specimens from patients with oral cavity squamous cell carcinoma (OSCC) and found that the coexistence of a specific mutation in the TERT promoter (-124 C>T) with a single nucleotide polymorphism (T/T genotype of the rs2853669) is associated with a poor prognosis in patients with early disease.

In early HNSCC, surgery has been shown to confer favorable outcomes depending on the primary site of the tumor. Laryngectomy has been considered the gold standard in advanced disease, either as primary or salvage therapy. However, several complications such as pharyngocutaneous fistula, are relatively common and lead to delays in adjuvant treatments and decline in quality of life. Using a very strict surgical protocol, an Italian group managed to reduce rates of this complication (Crosetti et al.). On the other hand, for very advanced disease involving the skull base that is stratified as T4b, carotid-sparing surgery or surgery including total carotidectomy is a therapeutic option that can lead to cure, albeit with serious sequelae such as carotid blowout. As meticulously discussed by Orlandi et al., every patient with carotid artery encasement is a unique case that requires management by multidisciplinary team including surgeons, radiotherapists, medical oncologists and interventional radiologists.

On the other hand, a transcriptionally active infection with Human Papilloma Virus (HPV), mainly HPV16, is a well-characterized contributing factor that is etiologically linked to a biologically distinct subgroup of oropharyngeal tumors with different clinical presentation and improved prognosis. Furthermore, patients with HPV-driven cancers do not commonly develop second primary tumors, which typically arise in the mucosa of the upper aerodigestive track following the accumulation of tobacco and alcohol-induced genetic alterations. In this context, Guarda et al. sought to assess the possibility of the development of synchronous and metachronous HPV-related oropharyngeal tumors by estimating the prevalence of a transcriptionally active HPV infection in the normal appearing mucosa next to and distant from the tumor. Interestingly, HPV was identified but not transcriptionally active in the cancer-free oropharyngeal tissue, indicating that the phenomenon of a field-cancerization prompted by HPV may not relevant in HPV-related oropharyngeal cancer.

Moreover, salivary gland carcinomas are relatively rare tumors that account for less than 5% of head and neck malignancies. A correction of an original review article discussing novel targets for advanced disease provides two precisely structured algorithms for adenoid-cystic and non-adenoid cystic carcinomas (Di Villeneuve et al.). Among all histological subtypes, mucoepidermoid carcinoma (MEC) is the most commonly encountered. Genetic studies may unravel biomarkers that can facilitate diagnosis and prognosis of MEC. Naakka et al. performed a miRNA and array-based gene expression analyses in 35 fresh frozen MEC samples and six normal salivary gland tissues and found that increased expression of mir-205 and mir-22 were associated with worse prognosis (). Interestingly, inhibition of these miRNAs in a MEC cell line resulted in reduced viability and invasion. Last, Piludu et al. highlighted the importance of MRI-based radiomics to enable differentiation of parotid lesions.

In conclusion, the present Research Topic has gathered several influential articles that sought to illustrate current knowledge regarding novel therapies and clinical/molecular biomarkers for head and neck carcinomas.

## Author contributions

All authors listed have made a substantial contribution to the work and approved it for publication.
